# Intra-Articular Giant Heterotopic Ossification following Total Knee Arthroplasty for Charcot Arthropathy

**DOI:** 10.1155/2013/472378

**Published:** 2013-09-15

**Authors:** Arata Nakajima, Shintaro Tsuge, Yasuchika Aoki, Masato Sonobe, Yoshifumi Shibata, Yu Sasaki, Koichi Nakagawa

**Affiliations:** ^1^Department of Orthopaedic Surgery, Toho University Sakura Medical Center, 564-1 Shimoshizu, Sakura, Chiba 285-8741, Japan; ^2^Department of Orthopedics and Rheumatology, Toho University Sakura Medical Center, 564-1 Shimoshizu, Sakura, Chiba 285-8741, Japan; ^3^Department of Orthopaedic Surgery, Toho University Omori Medical Center, 6-11-1 Omori-nishi, Ota-ku, Tokyo 143-8541, Japan

## Abstract

Although the Charcot arthropathy may be associated with serious complications, total knee arthroplasty (TKA) is the preferred choice of treatment by patients. This case report presents an 80-year-old man with intra-articular giant heterotopic ossification following loosening of femoral and tibial implants and femoral condylar fracture. He had undergone TKA because of Charcot neuropathy seven years ago and had been doing well since. Immediately after a left knee sprain, he became unable to walk. Because he had developed a skin ulcer on his left calf where methicillin-resistant *Staphylococcus aureus* was detected, we postponed revision surgery until the ulcer was completely healed. While waiting, intra-articular bony fragments grew larger and formed giant heterotopic ossified masses. Eventually, the patient underwent revision surgery, and two major ossified masses were carefully and successfully extirpated. It should be noted that intra-articular heterotopic giant ossification is a significant complication after TKA for neuropathic arthropathy.

## 1. Introduction

Neuropathic arthropathy was described by Jean Martin Charcot (1825–1893) as the progressive destruction of bone and soft tissues in a patient with peripheral neuropathy. Charcot also noted the relationship between syphilis and severe arthropathy in 1868 [1]. Diabetes mellitus is currently the most common cause of Charcot arthropathy, especially of the foot and ankle.

The diagnosis of Charcot arthropathy in the knee is rare. However, we can expect the increasing number of diabetic patients living longer due to improvements in medical treatment to show an increasing incidence of neuropathic arthropathy as a late effect of peripheral neuropathy. The optimal treatment for Charcot arthropathy of the knee is controversial. Some investigators [2–4] consider it as an absolute contraindication to total knee arthroplasty (TKA). Recently, however, several studies [5] have shown satisfactory results from TKA.

In general, TKA for Charcot arthropathy is associated with high incidence of such serious complications as periprosthetic fractures, aseptic loosening, dislocation of the patella and tibiofemoral joint, and deep infection [6–8]. However, due to excellent functional recovery compared with conservative therapy and arthrodesis, TKA should not be contraindicated.

We present a case report of an 80-year-old man with intra-articular, giant, heterotopic ossification and a femoral condylar fracture following loosening of femoral and tibial implants. To date, the occurrence of intra-articular, giant, heterotopic ossification as a significant complication after TKA for Charcot arthropathy has not been reported.

Our patient gave informed consent for data from his case to be submitted for publication.

## 2. Case Report

An 80-year-old man presented with a primary complaint of left knee pain for five months. He had undergone TKA with a posterior stabilized-type prosthesis (Scorpio Superflex, Stryker Orthopedics, Mahwah, NJ, USA) due to a neuropathic joint seven years ago and had been doing well since. Several days before his visit to our hospital, he had sprained his left knee and became unable to walk.

**Figure 1:**
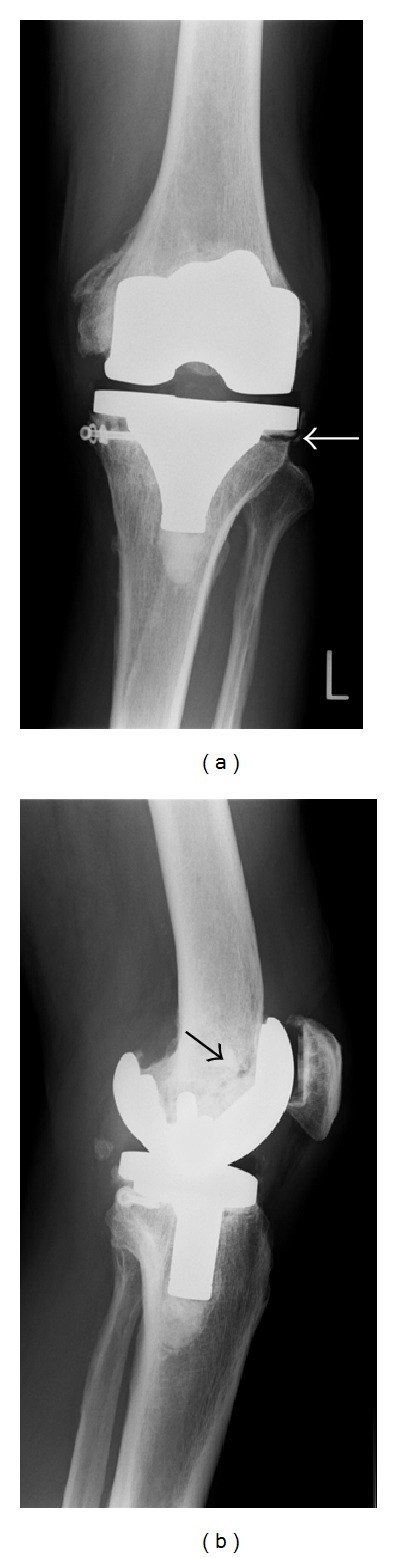
Radiographs of the left knee immediately after the patient sprained his knee and became unable to walk. An anteroposterior radiograph shows a radiolucency 1 mm in depth beneath the lateral aspect of the tibial component ((a), arrow), and the lateral view indicates a condylar fracture ((b), arrow).

**Figure 2:**
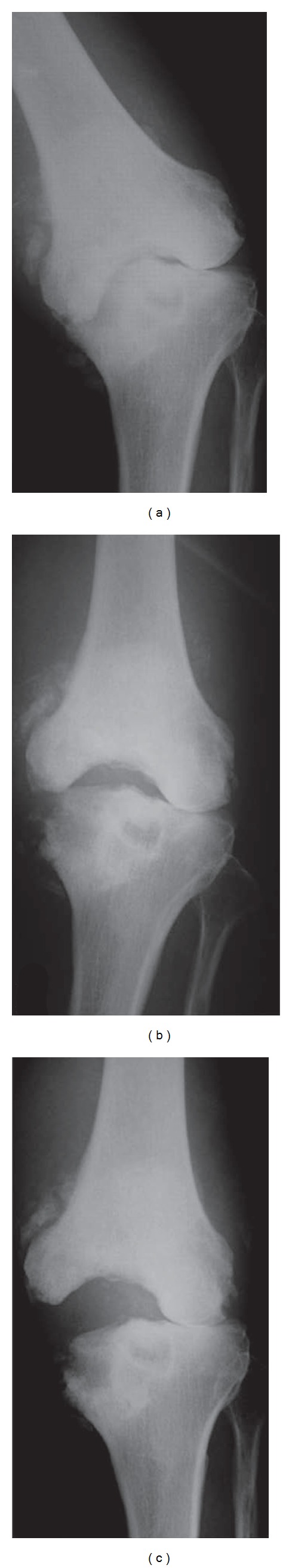
Stress radiographs of the left knee before the primary total knee arthroplasty (TKA) ((a) varus stress, (b) neutral, and (c) valgus stress). Severe joint destruction and instability are seen, indicating neuropathic arthropathy.

An anteroposterior radiograph showed a radiolucency 1 mm in depth beneath the lateral aspect of the tibial component (Figure 1(a), arrow), and the lateral view indicated a condylar fracture (Figure 1(b), arrow). We had planned to perform a revision TKA using a more strongly constrained-type knee prosthesis shortly thereafter, but this surgery had to be postponed because methicillin-resistant staphylococcus aureus (MRSA) was detected in a skin ulcer on his left calf. Although his peripheral leukocyte count was within normal limits (6.8 × 10^3^/*μ*L), his serum C-reactive protein level was elevated to 5.3 mg/dL (normal range <0.3 mg/dL) and synovial fluid bacterial cultures were negative. Stress radiographs for the left knee taken before the primary TKA showed severe joint destruction and instability (Figures 2(a)–2(c)), strongly suggesting neuropathic arthropathy. His hemoglobin A1c was 5.4%, and both the serological test for syphilis and Treponema pallidum hemagglutination test were negative. MRI images of the cervical and thoracic spine were normal, and syringomyelia was ruled out. However, he had been diagnosed wiyh primary amyloidosis and followed by neurophysicians. Peripheral nerve conduction velocity was severely delayed, suggesting neuropathic arthropathy due to amyloidotic polyneuropathy.

**Figure 3:**
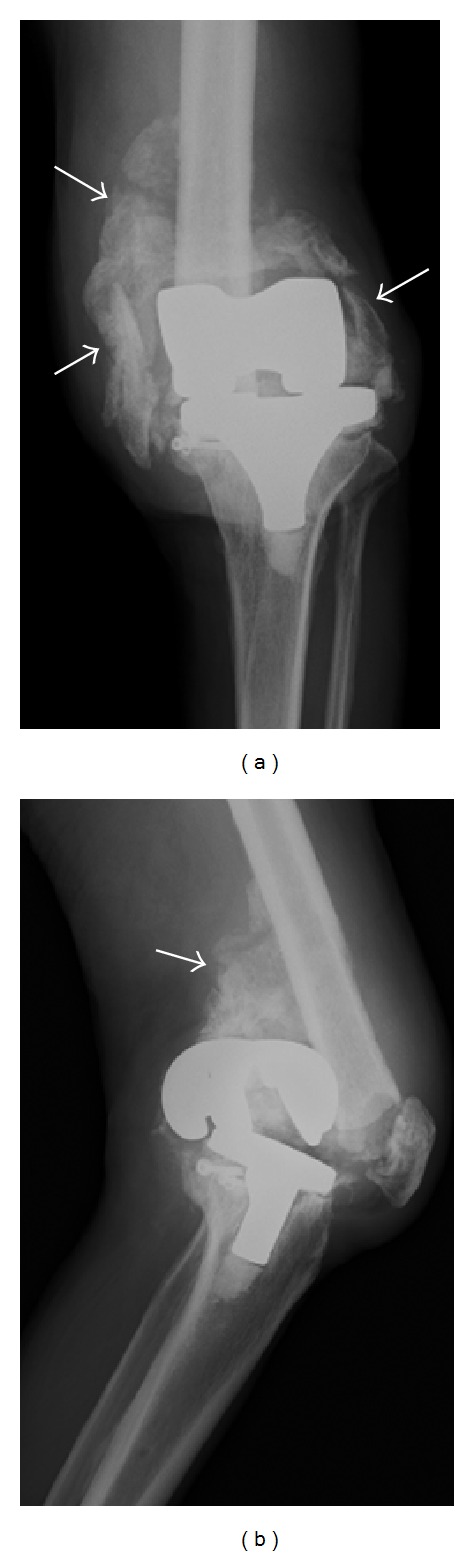
Radiographs of the left knee three months after the patient became unable to walk. Intra-articular bony fragments are enlarged and form giant ectopic ossified masses ((a), (b), arrows). Note that the femoral component is displaced and shifted posteriorly (b).

The skin ulcer did not completely heal for three months, during which time the patient was hospitalized with his left knee immobilized in a long-leg brace and administered with lipoprostaglandin E1. The patient did not understand our directions however, sometimes moving his left knee or walking around his room. As a result, the intra-articular bony fragments grew larger to form giant, ectopic ossified masses (Figures 3(a) and 3(b), arrows). To clarify the position of the popliteal artery with relationship to the ossified masses, we performed digital subtraction angiography (DSA) and confirmed that the popliteal artery was shifted posteriorly by the displaced femoral component and was very close to the ossified mass that appeared to adhere to the posterior capsule.

**Figure 4:**
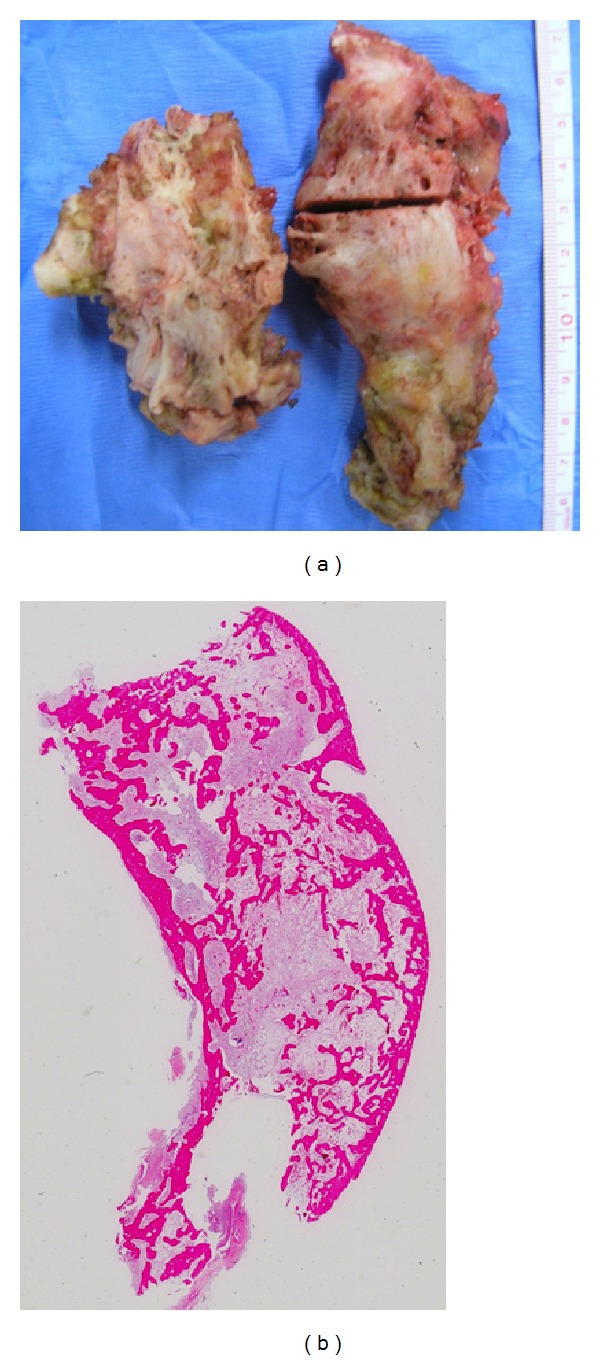
(a) Intra-articular giant ossified masses (12 × 4 and 8 × 4 cm in size) were firmly adherent to the posterior and medial capsules and required special care to extirpate. (b) Hematoxylin-eosin staining of the ossified mass showing typical structures of mature spongiosa.

The patient underwent revision surgery using a rotating hinged-type prosthesis (Modular Resection System, Stryker Orthopedics, Mahwah, NJ, USA). Two large ossified masses (12 × 4 and 8 × 4 cm in size, Figure 4(a)) were firmly adhered to the posterior and medial capsules but were successfully and completely extirpated. Hematoxylin-eosin staining of the ossified masses showed typical structures of mature spongiosa (Figure 4(b)). Eight weeks following surgery, the patient was permitted to walk with full weight-bearing on the left knee which was immobilized by a long-leg brace. The patient was discharged from the hospital 12 weeks after surgery.

Five months after surgery, however, the patient was readmitted to our hospital because of left foot gangrene and a periprosthetic fracture of the femur. His left leg was amputated above the knee, and he was discharged three months after amputation.

## 3. Discussion

Although the diagnosis of Charcot arthropathy in the knee is rare, there have been reports of TKA in knees with Charcot arthropathy [6–8]. Among these, Kim et al. and Parvizi et al. reported a relatively high incidence of serious complications following TKA with Charcot arthropathy [6, 7]. Kim et al. reported a high incidence of complications (nine of 19 knees, 47%) including dislocation of the patella and tibiofemoral joint, periprosthetic fractures, and rupture of the quadriceps tendon [6]. Parvizi et al. reported that six of their 40 cases (15%) had complications, including periprosthetic fractures, aseptic loosening, joint instability, and deep infection [7]. In our case, the patient had aseptic loosening with a condylar fracture, both likely to occur following TKA with Charcot arthropathy.

An unexpected complication in this case was the occurrence of giant, heterotopic ossification in the knee joint. Heterotopic ossification in a Charcot joint other than the knee has been reported in the elbow. Deirmengian et al. showed radiographs of neuropathic arthropathy of the elbow with dislocation, fracture fragmentation, and heterotopic ossification [9]. 

Since it has been demonstrated that abnormal bone metabolism is implicated in neuropathic arthropathy [10, 11], dynamic effects combined with altered bone metabolism may have promoted the development of heterotopic ossification in the knee joint. The metabolic control of bone is, at least in part, influenced by the nervous system. Potential transmitters for this influence include glutamate, calcitonin gene-related protein (CGRP), substance P, vasoactive intestinal peptide (VIP), pituitary adenylate cyclase activating polypeptide (PACAP), leptin, and catecholamines. These factors are known to be involved in bone formation, resorption, and remodeling [11], and imbalances in these factors may cause progressive joint destruction and/or giant ectopic ossification following implant loosening and intra-articular fractures.

Neurotrophic factors, such as brain-derived neurotrophic factor (BDNF) and nerve growth factor (NGF), are also potential factors influencing bone metabolism. In the rheumatic joint, a low density of sympathetic nerve fibers increased density of BDNF-positive cells in the synovium have been reported [12]. Koeck et al. reported that sympathetic nerve fibers were significantly lower in patients with Charcot foot compared to those with osteoarthritis (OA), and that the sympathetic nerve repellent factor semaphorin 3C was highly expressed in inflamed tissue of Charcot patients [13]. Considering these findings, BDNF and NGF, also known to be bone anabolic factors [14, 15], may be upregulated in Charcot arthropathy, leading to development of intra-articular giant ectopic ossification.

In condylar fractures complicated with implant loosening in the Charcot joint, as found in our case, bony fragments could develop into the giant, heterotopic ossified masses that would make the revision surgery more difficult. Therefore, surgeons should perform revision surgery as soon as possible following implant loosening or periarticular fractures in the Charcot joint.

Our concern is that there may have already been septic loosening of the implants, when the patient presented to our hospital following his knee sprain. His C-reactive protein serum levels were highly elevated (5.3 mg/dL) before the revision TKA, and bacterial cultures taken from the periprosthetic fracture site during amputation were positive for MRSA. These factors mean that we cannot preclude the possibility of existing bacterial infection in the patient's knee before the revision surgery. However, in addition to negative results for the synovial fluid cultures before the revision TKA, the elevated C-reactive protein serum levels returned to normal 19 days after the revision surgery, indicating that septic loosening prior to the condylar fracture did not occur. 

In conclusion, this case report is the first published account of the occurrence of giant, heterotopic ossification associated with an intra-articular fracture and implant loosening following TKA for neuropathic arthropathy. Although many other complications have been reported to follow TKA for Charcot arthropathy, it should be noted that intra-articular, giant, heterotopic ossification is a significant complication following TKA for Charcot arthropathy.
